# Physical Activity Levels and Recreational Participation Among Physiotherapists: A Cross-Sectional Correlational Study

**DOI:** 10.3390/jfmk10020164

**Published:** 2025-05-07

**Authors:** Constantin Ciucurel, George Mihail Man, Marilena Monica Tantu, Mariana Ionela Tudor, Georgeta Ionescu, Ana Catalina Tantu, Elena Ioana Iconaru

**Affiliations:** 1 Department of Medical Assistance and Physical Therapy, University Centre of Pitesti, National University of Science and Technology Politehnica Bucuresti, 110040 Pitesti, Romania; constantin.ciucurel@upb.ro (C.C.); george_mihail.man@upb.ro (G.M.M.); marilena.tantu@upb.ro (M.M.T.); mariana.tudor0610@upb.ro (M.I.T.); georgeta.ionescu82@upb.ro (G.I.); 2 Doctoral School in Medicine, University of Medicine and Pharmacy of Craiova, 200349 Craiova, Romania; catalina.tantu8@gmail.com

**Keywords:** physical activity, leisure time, lifestyle behaviours, occupational health, physiotherapists, active recovery

## Abstract

Background/Objectives: Healthcare professions impose high physical and mental demands, potentially affecting health. Despite advocating for active lifestyles, many physiotherapists do not meet recommended physical activity levels (PALs). This study explores physical activity patterns and recreational engagement among practicing physiotherapists. Methods: A cross-sectional correlational design examined the relationship between physical activity and recreational participation among 60 physiotherapists (38 females, 22 males, mean age 38.62 ± 9.78 years). Data were collected using the International Physical Activity Questionnaire—Short Form (IPAQ-SF) and the Pittsburgh Enjoyable Activities Test (PEAT). The analysis focused on bivariate correlations between PAL, energy expenditure (MET), recreational habits (PEAT score), and sociodemographic and anthropometric variables. Results: The IPAQ-SF results indicated that 38.3% of participants had a low PAL, 40.0% a moderate level, and 21.7% a high level. Average energy expenditure was 1927.62 ± 1479.73 MET-minutes/week. PEAT data indicated moderate engagement in recreational activities (mean score: 22.63 ± 7.76), with participants predominantly involved in low-intensity, passive leisure. Significant positive correlations were observed between PEAT scores and both MET values (Kendall’s tau-b = 0.669, *p* ≤ 0.01) and PALs (Kendall’s tau-b = 0.804, *p* ≤ 0.01), indicating that higher engagement in recreational activities is associated with greater energy expenditure and overall physical activity levels. Conclusions: Despite the physical demands of their profession, many physiotherapists report low activity levels. The positive correlations between physical activity, energy expenditure, and recreational engagement highlight the need for structured exercise to support both personal health and professional integrity. Future research should include larger samples and objective assessments.

## 1. Introduction

Healthcare professions are characterized by varying levels of physical and mental demands, which can lead to fatigue and stress. Moreover, physical activity in this context can become repetitive and may not always provide health benefits, potentially leading to discomfort and chronic impairments.

Studies on physical activity levels (PALs) among healthcare workers show contradictory results, likely due to the diversity of professions within the field [1]. For example, a recent study highlighted that a significant proportion of healthcare professionals have PALs below the World Health Organization’s recommended standards, with many showing low to moderate fitness levels in areas such as grip strength, upper body endurance, core strength, and cardiorespiratory fitness [2].

A revealing example is that of nurses, whose PAL varies depending on their roles, with lower levels observed among those in office-based positions [3]. Although their occupational activity mainly involves light-intensity tasks, occasional moderate-intensity demands persist in certain clinical roles, contributing variably to their overall physical exertion [4].

On the other hand, professions such as physiotherapy involve a more active workforce, with those working in public practice reporting higher levels of physical activity and participation compared to their counterparts in private practice [5]. However, despite being theoretical promoters of physical activity, physiotherapists do not always serve as personal role models, as many fail to meet the current physical activity recommendations [6]. Greater personal engagement in physical and leisure activities could enhance both their well-being and their role as credible advocates for active lifestyles.

The issue appears to originate as early as the training period of these professionals, as undergraduate physiotherapy students in some universities do not meet recommended PALs. In this population, high physical activity is associated with improved physical performance, while low activity levels are often linked to perceiving physical exertion as a barrier [7]. Some authors suggest that incorporating weekly physical activity monitoring into physiotherapy training would enhance students’ understanding of physical activity tracking and prepare them to use wearables to promote it in practice [8,9].

A large body of recent research has demonstrated the positive effects of leisure physical activity in mitigating burnout, reducing stress, and promoting overall health [10,11]. Moreover, effective management of leisure time has been shown to enhance professional performance and job satisfaction [12]. However, despite these findings, few studies have evaluated the relationship between professional PALs and engagement in recreational activities among individuals, let alone in the specific case of physiotherapists.

Despite the growing body of literature on occupational physical activity among healthcare workers, limited attention has been paid to the integration of structured physical activity and recreational engagement in the daily lives of physiotherapists themselves. Given their role as key promoters of movement, rehabilitation, and preventive care [13], it is paradoxical that physiotherapists may not consistently exemplify active lifestyles. Furthermore, discrepancies between professional recommendations and personal behaviour may undermine their credibility and long-term well-being.

This study aims to address this gap by exploring the PAL and recreational involvement among practicing physiotherapists, with an emphasis on identifying behavioural patterns and associated factors. Understanding these dynamics is essential both for informing professional health policies and self-care practices, and for reinforcing physiotherapists’ roles as credible advocates for physical activity in broader public health efforts.

Although participants in this study were recruited from a variety of professional contexts, the analysis did not focus on institutional comparisons. The diversity of settings was intended to enhance sample heterogeneity and provide a more generalizable perspective on physiotherapists’ physical activity behaviours. Prior research has highlighted that workplace characteristics, including organizational culture, clinical autonomy, and inter-professional collaboration, can meaningfully shape physiotherapists’ behaviours and professional engagement [14]. Nevertheless, systematic differences across working environments remain an important line of inquiry that warrants dedicated investigation in future studies.

## 2. Materials and Methods

### 2.1. Study Design

The study employed a cross-sectional correlational design to investigate the relationship between physical activity patterns and engagement in recreational activities among practicing physiotherapists. The primary aim was to identify significant correlations between PAL, energy expenditure, engagement in leisure activities, and sociodemographic and anthropometric variables. This approach seeks to uncover patterns that could inform strategies for promoting work–life balance and enhancing overall well-being in the professional context.

### 2.2. Study Population and Settings

The study included a sample of 60 practicing physiotherapists, aged between 24 and 61 years, 22 males and 38 females. Participants were recruited using a convenience sampling method from various healthcare settings, including hospitals, rehabilitation centres, and private practices, based on availability and willingness to participate in the study. Informed consent was obtained in writing from all participants prior to their inclusion in the study. Inclusion criteria were active employment as a physiotherapist at the time of the study, a minimum of six months of professional experience, and consent to participate in the research.

Exclusion criteria included individuals with any known physical condition that would impair their ability to engage in physical activity, those who were on extended leave or not actively practicing during the study period, and participants with insufficient data or incomplete questionnaires. The aim was to ensure a sample that accurately represented practicing physiotherapists actively engaged in their profession and physical activity.

Given that this is a correlational study, a power analysis of the correlation bivariate model was conducted using G*Power (version 3.1.9.4) to determine the required sample size for detecting a moderate correlation (r ≈ 0.35) with 80% power and an alpha level of 0.05 [15]. The analysis indicated that a sample size of 61 participants would be necessary. With 60 participants included in our study, we consider this sample size appropriate for the research objectives. Given the expected strength of the correlations, we anticipate that the observed correlations may exceed the moderate threshold, potentially enhancing statistical power.

### 2.3. Data Sources/Measurement

Data collection involved the administration of two instruments: the International Physical Activity Questionnaire—Short Form (IPAQ-SF) and the Pittsburgh Enjoyable Activities Test (PEAT), in printed format, following a brief standardized instruction. Sociodemographic (age, sex, years of practice—YPs) and anthropometric (body weight—W) data were also recorded.

The IPAQ-SF is a widely recognized instrument known for its good reliability and validity in measuring physical activity across different population groups [16]. It offers a systematic method for evaluating the frequency, duration, and intensity of physical activities across various domains, making it a valuable tool for both clinical practice and research applications [17,18]. The questionnaire assesses physical activity over the past week, considering factors such as work-related, transport, household, and leisure activities. It also calculates Metabolic Equivalent of Task (MET) values in minutes per week, as well as estimates of calorie expenditure, categorizing participants’ PALs into low, moderate, or high intensity [19]. The IPAQ-SF is licensed under the Creative Commons Attribution 4.0 International License (details available at: https://creativecommons.org/licenses/by/4.0/, accessed on 15 April 2025).

The Pittsburgh Enjoyable Activities Test (PEAT) is a validated 10-item instrument that assesses the frequency with which individuals have engaged in a variety of enjoyable activities (conducted alone or with others, in diverse settings, and encompassing both active and passive forms of leisure) over the past month. Respondents rate the frequency of each activity over the past month on a 5-point Likert scale (0 to 4), with higher scores reflecting more frequent engagement and greater enjoyment. The PEAT has been shown to be positively associated with improved physical and mental health outcomes, supporting the beneficial impact of recreational engagement on overall well-being. Furthermore, it has demonstrated acceptable internal consistency in previous research [20,21].

### 2.4. Variables and Statistical Methods

This study assessed several key variables, including age, sex, W, YPs, PAL, and MET values derived from the IPAQ-SF, as well as scores from the PEAT scale. Descriptive statistics, such as frequency distributions, means, and standard deviations (SDs), were calculated to summarize the sample’s characteristics.

To explore the relationships between the variables, a correlation matrix was created, assessing associations between age, sex, weight, YPs, PALs, MET values, and PEAT scores. The normality of the data was tested using the Shapiro–Wilk test to determine the appropriate correlation method. Bivariate correlations were computed using Kendall’s tau-b coefficient, appropriate for non-parametric data [22]. Statistical significance was determined based on a two-tailed p-value threshold of <0.05. Data analysis was conducted using IBM SPSS 26.0 software (IBM Corp., Armonk, NY, USA) [23].

## 3. Results

The following section reports the descriptive statistics of the study population and presents the results of the correlational analyses aimed at identifying significant associations between PALs and MET, recreational activity involvement, and selected demographic and professional variables.

### 3.1. Sample Characteristics

A total of 60 physiotherapists participated in the study, comprising 22 males (36.7%), and 38 females (63.3%), with a mean age of 38.62 ± 9.78 years (range: 24–61 years). The mean body weight was 68.58 ± 10.57 kg, and the average number of years of professional experience was 11.75 ± 7.53 years. All participants met the inclusion criteria and completed the full set of questionnaires.

Overall, the age and experience profiles of the sample indicate a professionally mature cohort, with most individuals situated in mid-career stages, potentially reflecting a stable engagement with the profession and accumulated clinical exposure.

### 3.2. Physical Activity Levels (IPAQ-SF)

Based on the IPAQ-SF data, among the total sample (n = 60), 38.3% of participants were classified as having low PALs, 40.0% as moderate, and 21.7% as high. No missing data were recorded for this variable, ensuring full response completeness. The mean MET-min/week was 1927.62 ± 1479.726. Both the categorical PAL distribution and the MET scores represent a global assessment of physical activity, capturing the combined effect of professional physical demands and leisure-time activity. Given the nature of physiotherapy as a physically interactive profession, these values provide insight into the overall physiological load experienced by practitioners. However, the distribution suggests that a significant proportion of participants may not reach optimal thresholds of health-enhancing physical activity, particularly when work-related exertion is not complemented by structured recreational exercise.

### 3.3. Recreational Activity Involvement (PEAT)

The mean PEAT score across the sample was 22.63 ± 7.76, indicating a moderate level of engagement in enjoyable recreational activities. The most frequently reported activity types included spending time unwinding at the end of the day (item 2, mean score per group 2.95 ± 0.87), involvement in hobbies (item 10, mean score per group 2.93 ± 0.66), and being in outdoor settings such as gardens, parks, countryside (item 8, mean score per group 2.77 ± 0.67). These activities, while subjectively enjoyable, appear to involve limited physical effort and are predominantly characterized by low-intensity, passive leisure behaviours.

### 3.4. Correlational Analysis

To determine the appropriate statistical method for examining associations among the study variables, we first assessed the distribution of continuous variables (age, W, YPs, MET, and PEAT scores) using the Shapiro–Wilk test for normality. The results indicated that only the PEAT score followed a normal distribution, while all other variables deviated from normality. Considering the non-normal distribution of most variables, and the ordinal or categorical nature of others (sex and PAL), the Kendall’s tau-b correlation coefficient was selected for bivariate analysis. This method is well-suited for datasets including ordinal or non-normally distributed continuous variables and provides a robust measure of monotonic association [24].

All variables were included in a single correlation matrix, allowing a comprehensive exploration of relationships between sociodemographic and anthropometric characteristics, physical activity indicators, and recreational engagement levels. Table 1 presents the full matrix of Kendall’s tau-b correlation coefficients, along with the corresponding statistical significance (*p*-values) for each bivariate association.

In relation to the objectives of our study, statistically significant positive correlations were observed between PEAT scores and MET values (Kendall’s tau-b = 0.669, *p* ≤ 0.01; strong correlation), as well as between PEAT scores and PALs (Kendall’s tau-b = 0.804, *p* ≤ 0.01; very strong correlation). These findings suggest that higher engagement in recreational activities is strongly associated with both increased energy expenditure and elevated PALs. This implies that individuals who engage more frequently in enjoyable leisure activities tend to exhibit higher levels of physical exertion, as reflected in both their MET scores and physical activity profiles, underscoring the reciprocal relationship between recreational participation and overall physical fitness.

Specifically, the robust correlation between PEAT scores and MET values, coupled with the even stronger correlation with PALs, suggests that individuals with greater engagement in recreational activities tend to exhibit superior overall physical fitness, which may positively influence their physical well-being and capacity for daily activities.

**Figure 1:**
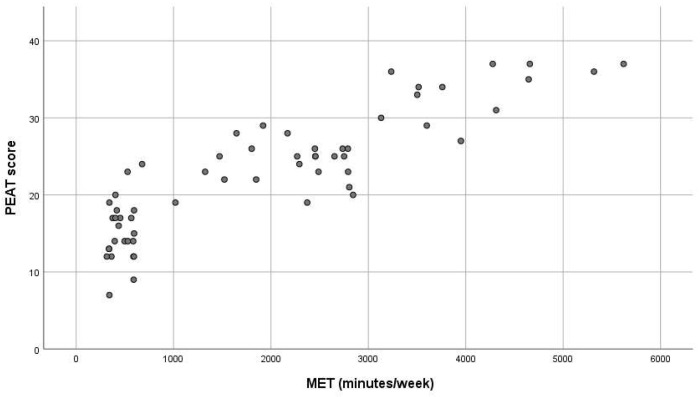
Relationship between PEAT scores and MET values (scatterplot).

**Figure 2:**
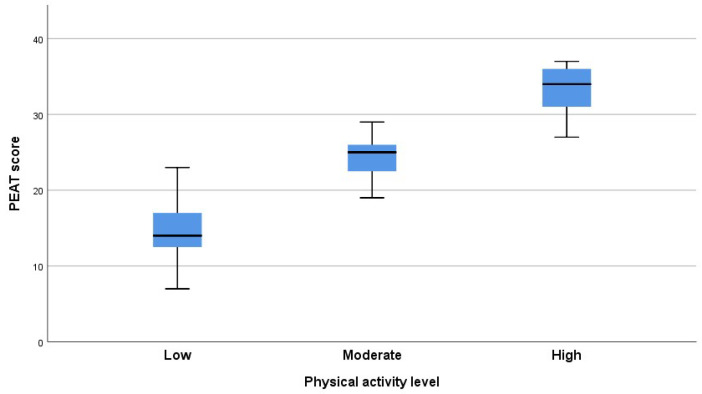
Distribution of PEAT scores across PALs (boxplot).

To visualize these relationships, a scatterplot (Figure 1) was used to represent the relationship between PEAT scores and MET values (continuous variables), while a boxplot (Figure 2) was employed to illustrate the distribution of PEAT scores across (an ordinal variable), considering the distinct nature of these variables.

Figure 1 illustrates the relationship between PEAT scores and MET values, highlighting a positive association. Visual inspection of the scatterplot suggests that higher levels of physical activity, as reflected by MET values, are generally associated with elevated PEAT scores, in line with the observed correlation (τ = 0.669, *p* < 0.001).

As observed in Figure 2, the median PEAT scores were 14 for participants with low PAL, 25 for those with moderate PAL, and 34 for those with high PAL. The corresponding interquartile ranges (IQRs) were 5, 4, and 6, respectively, indicating slightly greater variability in leisure activity engagement among participants with high PAL.

Other statistically significant correlations were observed between PAL and MET (Kendall’s tau-b = 0.811, *p* ≤ 0.01), age and YPs (Kendall’s tau-b = 0.88, *p* ≤ 0.01), and sex and W (Kendall’s tau-b = −0.61, *p* ≤ 0.01). The strong positive association between PAL and MET is theoretically expected, as the two variables are computationally interrelated within the same assessment framework, thus supporting the internal consistency and coherence of the physical activity estimation. The correlation between age and YPs reflects a natural and anticipated trajectory, whereby years of professional experience increase proportionally with age. In contrast, the inverse correlation between sex and W—considering the coding used (1 = male, 2 = female)—indicates higher body weight values in male participants, a result consistent with documented sex-related differences in body composition.

All other bivariate correlations did not reach statistical significance, indicating the absence of meaningful monotonic associations between the remaining pairs of variables in the context of this sample.

## 4. Discussion

This study aimed to explore physical activity patterns among physiotherapists, as reflected by PAL and MET, and to examine their associations with recreational engagement and relevant sociodemographic and anthropometric variables. This research is particularly relevant, given the increasing recognition of the importance of physical activity in maintaining both physical and mental health, especially among healthcare professionals. The findings of this study contribute to a growing body of literature addressing the PALs and recreational activity engagement of physiotherapists, a profession that is expected to both promote and exemplify active lifestyles.

An analysis of the study sample reveals that it comprises physiotherapists, a professional cohort likely to exhibit distinct patterns of physical activity and leisure behaviours due to their specialized training and occupational demands. The sample consisted of 60 physiotherapists, with a gender distribution of 63.3% females and 36.7% males, and a mean age of 38.62 ± 9.78 years (range: 24–61 years). These demographic characteristics are representative of a mid-career cohort, suggesting a sample with substantial professional experience and clinical exposure. There is substantial evidence that physiotherapists, particularly those with extensive experience, recognize the importance of promoting exercise in therapy [25], and it is reasonable to expect them to apply similar attitudes in their personal lives.

Based on the IPAQ-SF data, the physical activity regime within the sample indicate a mixed profile, with 38.3% of participants reporting low activity levels, 40.0% moderate, and 21.7% high activity levels. Despite the demands of their physically interactive profession, a significant proportion of physiotherapists may not meet optimal health-enhancing activity thresholds, particularly when professional exertion is not supplemented by regular recreational exercise. However, the predominance of moderate PALs among participants aligns with findings from other studies, which highlight similar patterns of physical activity among physiotherapists, associated with a high perceived quality of life [26].

In relation to this topic, the literature reveals a broad spectrum of findings, with physical activity regime among physiotherapists varying significantly across studies. Some studies indicate that physiotherapists meet or exceed health guidelines, positioning them as role models for promoting physical activity to both patients and colleagues [5]. However, other research highlights a prevalence of suboptimal PALs raising concerns about the potential impact this may have on their professional role, including the therapeutic relationship with patients [6]. This variation calls for further exploration of the factors influencing physical activity behaviours in physiotherapists and their implications for patient care.

The essence of the physiotherapy profession lies in both technical expertise and the ability to lead by example. Physiotherapists serve as both providers of therapeutic interventions and guides for their patients in the recovery and functional optimization process [27]. Physical activity is therefore an expression of professional commitment, extending beyond just a recommendation for patients.

Another parameter investigated in our study was recreational activity involvement, assessed using the PEAT scale. The mean PEAT score for the group was 22.63 ± 7.76, indicating moderate engagement in enjoyable recreational activities. The most frequently reported activities were relaxation, hobbies, and spending time in nature. Although these are generally perceived as subjectively enjoyable and often involve low-intensity, passive leisure behaviours, certain forms of hobbies or nature-related activities may also include moderate levels of recreational physical engagement, depending on individual preferences and contexts. However, the assessment provided by the PEAT scale is qualitative in nature, relying on the ranking of leisure activities and offering only a general tendency rather than a precise quantification of recreational engagement [28]. It serves as an indicative measure, capturing the subjective dimension of leisure rather than its intensity or frequency [29].

It is worth noting that the mean PEAT score observed in our sample closely mirrors the population-level averages reported by the scale’s author, who identified a mean score of approximately 22 across a large adult cohort, with the majority of individuals (around 70%) scoring between 16.5 and 27.5 [20]. Additionally, recreational engagement among physiotherapists may be viewed in relation to broader contextual factors such as socioeconomic status and educational attainment, which is often associated, at both individual and community levels, with a tendency toward improved self-rated health and well-being across various population groups [30,31].

The balance between active and passive forms of recreation remains a subject of on-going discourse, particularly in light of evidence suggesting that physically engaging leisure activities may confer greater health benefits compared to predominantly sedentary modes of relaxation [32]. The present findings align with the previous literature suggesting that, as individuals age, they tend to engage more frequently in passive recreational activities, whereas active leisure participation continues to be a significant predictor of life satisfaction in later life, independent of confounding variables [33].

The final analysis conducted was correlational in nature. The results revealed significant correlations between PEAT scores and MET values (Kendall’s tau-b = 0.669, *p* ≤ 0.01), as well as between PEAT scores and PALs (Kendall’s tau-b = 0.804, *p* ≤ 0.01). These findings suggest that greater involvement in recreational activities is strongly associated with increased energy expenditure and higher PALs highlighting the relationship between leisure activity participation and physical activity outcomes.

The analysis should be nuanced, as while physiotherapists engage in physically demanding work, their leisure activities tend to be more sedentary, focused on relaxation and recovery. This suggests that the physical activity they perform professionally may be compensated by passive recreational behaviours during their free time.

In this context, research indicates that physical inactivity is often influenced by specific life stages or cycles [34], particularly those marked by significant transitions or challenges, which may lead individuals to spend more time in sedentary activities, even when they maintain an active professional lifestyle [35]. This shift highlights the importance of balancing active and passive recreational pursuits. For physiotherapists, incorporating active leisure activities could promote better overall health and well-being, serving as an example for patients and improving their own physical and mental resilience.

Given the systemic nature of physical inactivity, interventions must integrate behavioural theories across multiple levels to address these challenges in both professional and personal contexts [36]. Physiotherapists, equipped with the necessary knowledge and skills, are well-positioned to provide physical activity advice during routine consultations [37]. Furthermore, leading by personal example is seen as an essential component of effective lifestyle interventions. By maintaining their own physical fitness, physiotherapist position themselves as credible role models, uniquely equipped to motivate and guide their patients toward adopting healthier, more active lifestyles [38].

These findings underscore the importance of developing targeted strategies aimed at promoting physiotherapists’ health and sustaining high levels of professional performance. Given their dual role as healthcare providers and behavioural exemplars, physiotherapists would benefit from structured institutional programs that integrate workplace physical activity, stress reduction techniques, and active leisure time planning. Strategies such as periodic wellness evaluations, personalized activity prescriptions, and organizational support for recreational engagement (e.g., on-site fitness sessions or incentivized participation in physical activity programs) may enhance both physical well-being and job satisfaction. Embedding such initiatives into the professional culture could reinforce the alignment between personal health behaviours and therapeutic advocacy, strengthening the credibility and efficacy of physiotherapists as role models.

In summary, this study highlights the complex interplay between professional physical demands and leisure-time behaviours among physiotherapists. While their occupational roles involve substantial physical activity, this appears to be counterbalanced by predominantly sedentary recreational habits, likely reflecting a need for psychological recovery. The observed associations between recreational engagement and physical activity parameters (MET and PAL) emphasize the relevance of lifestyle patterns beyond the workplace. As credible health promoters, physiotherapists are uniquely positioned to influence patient behaviour, and adopting active leisure practices themselves may enhance both their effectiveness and professional authenticity.

This study presents several limitations that should be acknowledged. The sample size was relatively small and limited to a specific professional group, which may affect the generalizability of the findings to broader populations or other healthcare professions. Additionally, the cross-sectional design restricts the ability to infer causality between PALs and recreational behaviour. The use of self-reported instruments, such as the IPAQ-SF and PEAT, introduces potential recall and social desirability biases. Moreover, the qualitative nature of the PEAT scale limits the precision of measuring the intensity and frequency of leisure activities. Future research would benefit from longitudinal designs and objective monitoring tools to validate and expand upon these findings. Furthermore, the absence of multivariate analysis precluded statistical control for potential confounding variables, which may have influenced the observed associations. This represents an additional limitation to the internal validity of the study. Future studies should incorporate multivariate and inferential models (e.g., regression or ANOVA) to better account for potential confounding and strengthen the explanatory power of the findings.

## 5. Conclusions

Despite the physically demanding nature of their profession, a significant proportion of physiotherapists engage insufficiently in health-enhancing physical activity. The significant positive correlations observed between recreational habits, PAL, and energy expenditure indicate the relationship between leisure-time activities and physical activity outcomes. Nevertheless, the persistence of predominantly passive recreational behaviours among respondents may reflect a compensatory response to occupational fatigue, potentially limiting the cumulative benefits of regular physical activity beyond work-related exertion.

To fulfil their role as health promoters, physiotherapists must model active lifestyles beyond occupational demands. In addition, implementing specific strategies to promote active leisure and conducting long-term follow-up studies would contribute to sustaining physiotherapists’ health and professional performance. Promoting structured, enjoyable physical activity is essential for both personal health and professional credibility. Future studies should incorporate larger cohorts and objective measurements to better characterize these behavioural trends.

## Figures and Tables

**Table 1:** Bivariate correlation matrix (Kendall’s tau-b) for study variables.

Variable	Sex	Age (years)	W (kg)	YPs (years)	IPAQ-SFPAL	MET (min/week)	PEAT Score
Sex	1	−0.044 CI: −0.276, 0.192	−0.61 ** CI: −0.694, −0.514	0.001 CI: −0.238, 0.249	0.018 CI: −0.254, 0.260	0.025 CI: −0.220, 0.240	0.03 CI: −0.229, 0.263
Age (years)	−0.044 CI: −0.276, 0.192	1	0.036 CI: −0.192, 0.258	0.88 ** CI: 0.816, 0.924	0.077 CI: −0.136, 0.291	0.094 CI: −0.123, 0.282	0.058 CI: −0.154, 0.274
W (kg)	−0.61 ** CI: −0.694, −0.514	0.036 CI: −0.192, 0.258	1	0.006 CI: −0.217, 0.219	0.076 CI: −0.155, 0.301	0.077 CI: −0.113, 0.262	0.026 CI: −0.161, 0.205
YPs (years)	0.001 CI: −0.238, 0.249	0.880 ** CI: 0.816, 0.924	0.006 CI: −0.217, 0.219	1	0.11 CI: −0.104, 0.317	0.107 CI: −0.097, 0.297	0.087 CI: −0.109, 0.288
IPAQ-SF PAL	0.018 CI: −0.254, 0.260	0.077 CI: −0.136, 0.291	0.076 CI: −0.155, 0.301	0.11 CI: −0.104, 0.317	1	0.811 ** CI: 0.766, 0.831	0.804 ** CI: 0.748, 0.836
MET (min/week)	0.025 CI: −0.220, 0.240	0.094 CI: −0.123, 0.282	0.077 CI: −0.113, 0.262	0.107 CI: −0.097, 0.297	0.811 ** CI: 0.766, 0.831	1	0.669 ** CI: 0.561, 0.762
PEAT score	0.03 CI: −0.229, 0.263	0.058 CI: −0.154, 0.274	0.026 CI: −0.161, 0.205	0.087 CI: −0.109, 0.288	0.804 CI: 0.748, 0.836	0.669 ** CI: 0.561, 0.762	1

Note—W: weight; YPs: years of practice; PAL: physical activity level; IPAQ-SF: International Physical Activity Questionnaire—Short Form; MET: Metabolic Equivalent of Task; PEAT: Pittsburgh Enjoyable Activities Test; CI: confidence interval; **: correlation is significant at the 0.01 level (two-tailed).

## Data Availability

The data are available on request from the corresponding author. All data relevant to the study are included in the article.
